# The Impact of Hypomagnesemia on the Long-Term Evolution After Kidney Transplantation

**DOI:** 10.3390/nu17010050

**Published:** 2024-12-27

**Authors:** Ioana Adela Ratiu, Corina Moisa, Luciana Marc, Nicu Olariu, Cristian Adrian Ratiu, Gabriel Cristian Bako, Anamaria Ratiu, Simona Fratila, Alin Cristian Teusdea, Mariana Ganea, Mirela Indries, Lorena Filip

**Affiliations:** 1 Faculty of Medicine and Pharmacy, University of Oradea, 1st December Square 10, 410073 Oradea, Romania; ioana.ratiu@didactic.uoradea.ro (I.A.R.); ratiu_cristian@uoradea.ro (C.A.R.); gabriel.bako@uoradea.ro (G.C.B.); sfratila@uoradea.ro (S.F.); mganea@uoradea.ro (M.G.); mirela.indries@uoradea.ro (M.I.); 2 Nephrology Department, Emergency Clinical Hospital Bihor County, 410087 Oradea, Romania; 3 Division of Nephrology, Department of Internal Medicine II, “Victor Babeș” University of Medicine and Pharmacy, 300041 Timișoara, Romania; marc.luciana@umft.ro (L.M.); nicu.olariu@umft.ro (N.O.); 4 Research in Nephrology and Vascular Disease, Faculty of Medicine, “Victor Babeș” University of Medicine and Pharmacy, 300041 Timișoara, Romania; 5 Faculty of Dentistry, University of Medicine and Pharmacy “Iuliu Hatieganu” Cluj-Napoca, Victor Babeș Street 8, 400012 Cluj-Napoca, Romania; ratiu.anamaria@elearn.umfcluj.ro; 6 Dermatology Department, Emergency Clinical Hospital Bihor County, Republicii Street 37, 410167 Oradea, Romania; 7 Faculty of Environmental Protection, University of Oradea, 26th Gen. Magheru Avenue, 410087 Oradea, Romania; atedea@uoradea.ro; 8 Faculty of Pharmacy, University of Medicine and Pharmacy “Iuliu Hatieganu” Cluj-Napoca, Victor Babeș Street 8, 400012 Cluj-Napoca, Romania; lfilip@umfcluj.ro; 9 Academy of Romanian Scientists (AOSR), 3 Ilfov Street, 050044 Bucharest, Romania

**Keywords:** magnesium, kidney transplantation, infections, immunosuppression

## Abstract

Background/Objectives: Magnesium plays a crucial role in immune function, influencing immunoglobulin synthesis, antibody-dependent cytolysis, and other immune processes. In renal transplant patients, magnesium deficiency is primarily induced by calcineurin inhibitor treatment, through the reduction of magnesium transporter proteins in the renal tubules, leading to magnesium loss. Methods: To assess the correlation between serum magnesium levels and the long-term outcomes of renal graft and transplant recipients, we conducted a retrospective study on 87 patients who have had a transplant for more than 5 years, a period considered immunologically stable. We evaluated laboratory parameters such as glycemia, creatinine, total protein, and C-reactive protein (CRP), as well as demographic data, primary kidney disease, donor type, comorbidities, and infection incidence. Results: This study revealed clinical stability at over 5 years post-transplantation, with no significant differences between the 5–15 and over-15-years groups with regard to major comorbidities, except for HCV infection (*p* = 0.018). Reduced magnesium levels were associated with impaired renal function (*p* = 0.017) and inflammatory syndrome (*p* = 0.012). Viral infections were correlated with living donor grafts (*p* = 0.05), hypoproteinemia, and decreased eGFR (estimated glomerular filtration rate), while bacterial infections, namely urinary tract infections (UTIs), were linked to reduced eGFR (*p* = 0.05, *p* = 0.046). Female patients with hypomagnesemia had a higher incidence of recurrent UTIs (*p* = 0.03). Conclusions: Hypomagnesemia correlates with increased infection risk in patients who received a renal transplant more than 5 years ago but does not significantly impact glycemic control or cardiovascular health.

## 1. Introduction

Magnesium, the second most abundant cation in the body, is regulated by complex mechanisms involving intestinal absorption and renal excretion. Hypomagnesemia is associated with chronic inflammation, which affects cardiovascular health, metabolic balance, obesity, oncogenesis, and osteoporosis [1,2,3]. In certain situations [4,5,6,7,8,9], this has fatal consequences [10,11]. Animal studies have shown that hypomagnesemia activates leukocytes and macrophages that release inflammatory cytokines and generate free radicals [10].

Magnesium metabolism involves active transcellular uptake via the transient receptor potential melastatin 6 (TRPM6) channel, passive paracellular diffusion in the intestines, and reabsorption predominantly in the Henle loop. Urinary magnesium loss, of about 5% of the entire amount filtered, is influenced by various intestinal or renal pathways, with homeostasis being maintained by continuous feedback between absorption and excretion [11]. If Mg intake is low, urinary elimination decreases and vice versa; thus, excessive or subliminal serum magnesium values imply the exceedance of the physiologic mechanisms of bodily self-regulation [12].

Hypomagnesemia is correlated with the presence of other ionic imbalances, particularly with serum potassium. Reduced magnesium levels impair ATP-ase activity, including ATP-dependent K+ channels at the tubular level, leading to hypokalemia. The therapeutic implication lies in the need to correct magnesium levels in patients with hypokalemia alongside specific corrective measures.

Magnesium deficiency is also correlated to an increase in intracellular Ca^2+^ through the activation of L-type calcium channels and N-methyl-D-aspartate (NMDA) receptors. As a result, nuclear factor-κB (NFκB) (including TNF-α and IL-1β) increases, as well as the amount of substance P at the neuronal level, resulting in consequent oxidative nerve injury [13].

**Figure 1:**
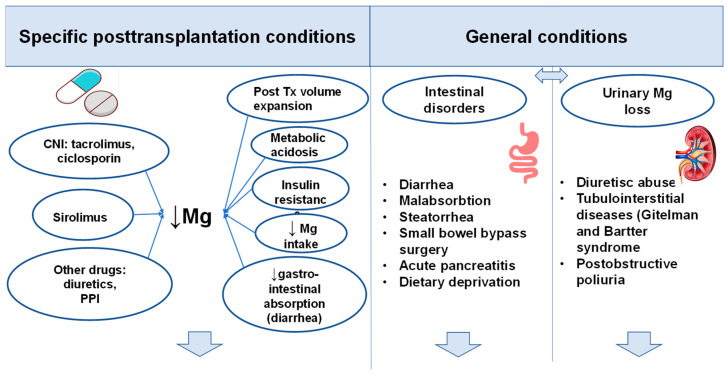
Etiology of Hypomagnesemia after a Kidney Transplant (↓ Mg-low level Mg).

The issue is even more complex for kidney transplant recipients (KTRs). Figure 1 illustrates the potential causes of post-transplant hypomagnesemia in this group of patients.

In kidney transplant recipients (KTRs), magnesium deficiency is primarily induced by treatment with calcineurin inhibitors (CNIs). Compared to ciclosporine, tacrolimus causes hypomagnesemia five times more frequently [14]. The mechanism by which CNIs induce hypomagnesemia is inadequately explored; however, it is likely attributed to the decreased production of epidermal growth factor (EGF), which inhibits the activation of TRPM6 [15].

Sirolimus is less commonly used in post-transplant immunosuppressive regimens. Animal studies have demonstrated a similar effect on inducing urinary magnesium loss as seen with CNIs. However, m-TOR inhibitors contribute to magnesium loss through a different mechanism involving the inhibition of Na-K-Cl co-transporter 2 expression in the loop of Henle [16].

**Figure 2:**
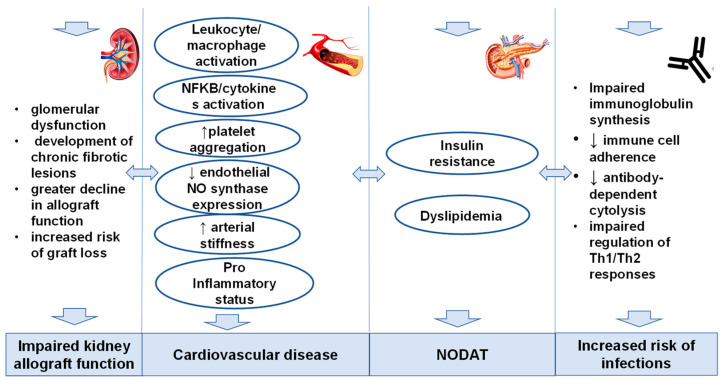
The Impact of Hypomagnesemia after Kidney Transplantation (Legend: NODAT—new onset of diabetes mellitus after transplantation; NFKB—Nuclear factor-κB, ↑ high lewel, ↓ low level).

Hypomagnesemia has a significant in KTRs (Figure 2).

Data from the literature indicates a bidirectional relashionship between hypomagnesemia and renal function. Impairment of renal graft function results in increased magnesium loss due to tubular fibrosis; conversely, reduced magnesium levels negatively influence graft function [17,18].

Hypomagnesemia has multiple additional consequences in KTRs. The occurrence of new-onset diabetes after transplant (NODAT) is extremely common, hypomagnesemia being one of the contributing causes [19,20].

Similar to the general population, Mg induces NODAT through direct effects on transmembrane glucose transport and glucose oxidation, modulation of insulin secretion at the pancreatic level, and alteration of insulin receptor response. Given the numerous other factors associated with the onset of NODAT, it is challenging to assert that hypomagnesemia is an independent risk factor for this condition, although most studies support this hypothesis. Animal studies and clinical trials with non-transplant diabetic patients indicate that magnesium supplementation improves glucose tolerance and insulin sensitivity [21,22].

Hypomagnesemia is correlated with the onset or worsening of cardiovascular pathology through both humoral and cellular inflammation [6]. Hypomagnesemia plays a role in reducing leukocyte and macrophage activity, decreasing nitric oxide (NO) synthesis, increasing CRP and IL-1 levels, cytokine hypersecretion, and enhanced platelet aggregability—conditions that are further exacerbated in patients with chronic kidney disease (CKD) [23,24]. All these factors contribute to hypertension through increased vascular stiffness and worsening cardiovascular conditions via inflammation and endothelial dysfunction.

A decrease in serum magnesium levels is associated with a higher risk of infections [25]. In KTRs, the risk of infection is predominantly a direct result of immunosuppressive therapy. Despite constant efforts to achieve optimal therapies with immunosuppressants, the risk of toxicity, as well as underdosing, means that bacterial, viral, fungal, or parasitic infections are an inevitable event along the post-transplant course. Therefore, correcting all additional factors that contribute to the heightened infection risk, including managing magnesium deficiency, is essential after transplantation. It is well-known that hypomagnesemia appears early after transplantation and persists for approximately two years before serum magnesium levels tend to normalize. However, there are cases in which hypomagnesemia persists for much longer, with the consequences yet to be fully evaluated.

Considering the numerous implications of serum magnesium levels in post-transplant pathology, as well as the limited number of published studies in this field, this study aims to evaluate the risks associated with hypomagnesemia in patients in the late post-transplant period, specifically those more than 5 years after receiving a renal graft, with a focus on infectious complications. There is a lack of data in the literature concerning this issue in patients who are over five years post-transplant. Additionally, this study analyzes how magnesium supplementation influences long-term outcomes in renal transplant recipients.

## 2. Materials and Methods

### 2.1. Study Design and Patient Characteristics

This retrospective, observational, single-center study involves kidney transplant recipients from the Nephrology Clinic of one of the largest hospitals in western Romania. The follow-up period was 12 months (1 January to 31 December 2023). This study was approved by the Ethics Committee of the Bihor County Clinical Emergency Hospital (approval no. 7701/02.03.2023) and the Ethics Committee for access to the KTRs database (approval no. 8847/13.03.2023). The exclusion criteria were: (i) age under 18 years and (ii) the presence of another type of organ transplant.

To assess the homogeneity of patient characteristics, the patients were divided into two groups: (i) KTRs with a post-transplant period of 5 to 15 years, and (ii) KTRs with a post-transplant period of over 15 years. The reason for choosing these intervals is the evolution of kidney grafts. As the average survival time of a kidney graft is 20 years, signs of renal dysfunction with increasing immune deficiencies may be expected 15 years after transplant. The first group included 51 patients, while the second included 36 patients.

This study has three steps:

- Comparative evaluation between the two groups, regarding demographic data (age and sex), underlying kidney disease, donor type, immunosuppressive medication, associated conditions, and presence and type of infections, as well as the use of magnesium supplements (including type and dosage).
- The evaluation of the laboratory tests performed for the two patient groups included assessments of creatinine levels, eGFR, blood glucose, C-reactive protein, total proteins, and serum magnesium. The characteristics of KTRs with hypomagnesemia, including laboratory tests, were then compared with KTRs with normal serum magnesium levels. Laboratory tests were performed using an Alinity analyzer (ABBOTT, Hannover, Germany), which uses the turbidimetric assay for C-reactive protein (Alinity AC03944) and spectrophotometry for the other measurements. For the determination of total protein, the electric field migration/densitometry method was used with the Hydrasys analyzer SEBIA, Lisses, France). The GFR was estimated using the CKD-EPI 2021 formula, according to KDIGO 2024 guidelines.
- We recorded the occurrence of infections requiring hospitalization from the GP files, irrespective of their location and type (bacterial, viral, or fungal), together with related cardiovascular conditions. We subsequently identified factors associated with the incidence of infections.

### 2.2. Type of Infections

Severe bacterial infections requiring hospitalization, regardless of the organ affected, were recorded. In this study, urinary tract infections and respiratory tract infections were investigated. The presence/activity of the following viruses was also assessed: herpes simplex virus, herpes zoster, HBV and HCV, CMV, papilloma, polyoma, and COVID-19.

### 2.3. Statistical Analysis

We performed statistical correlations to identify factors associated with reduced serum Mg levels and to determine whether magnesium deficiency increases the risk of post-transplant infections. Data were analyzed using R version 4.3.0. Shapiro–Wilk was employed for normality assessment, followed by the Wilcoxon test for non-parametric comparisons. For cross-tabulation, we decided to use the Fisher test, rather than chi-squared as it was often the case that some table cells had a low count.

## 3. Results

### 3.1. General Data

The relationship between post-transplant duration in each group (5–15 years and over 15 years, respectively) on one side, and patient characteristics, laboratory test results, infection type, and infection count on the other side, is presented in Table 1.

The mean age of the patients included in this study was 50.05 ± 11.94 years, with no significant age or gender differences between groups (*p* = 0.3 and *p* = 0.19, respectively). Male patients constituted 52.97% of the cohort. The only significant associations were observed with corticosteroid treatments (*p* = 0.026) and HCV infections (*p* = 0.018).

Deceased donor kidney allograft (DDKA) accounted for 79.3% of cases. Transplant recipients from a living donor had a statistically significant higher incidence of hypomagnesemia in the group with over 15 years post-transplant (72.72%) as compared to 5–15 years post-transplant (25% of cases).

Given the homogeneity of the groups, we decided to conduct a more in-depth statistical evaluation without differentiating based on the time elapsed since kidney transplantation (Tx).

### 3.2. Hypomagnesemia

According to the data presented in Table 2, no statistically significant differences were noticed regarding age (*p* = 0.214), sex (*p* = 0.82), or time elapsed since Tx (*p* = 0.252) between KTRs with or without hypomagnesemia. Underlying kidney disease (UKD) did not significantly influence the incidence of post-transplant hypomagnesemia (*p* = 0.26).

Hypomagnesemia correlated significantly with elevated CRP levels (*p* = 0.0012), higher creatinine (*p* = 0.028), and lower eGFR (*p* = 0.017).

Among the comorbidities potentially correlated with magnesium deficiency, NODAT was observed at a lower rate in KTRs with hypomagnesemia, though without statistical significance. In the 5–15 years post-transplant group of KTRs, NODAT was present in 13.72% of cases, among whom 57.14% had magnesium deficiency. At over 15 years post-Tx, the incidence of NODAT is 13.89%, with hypomagnesemia in 60% of these KTRs. Arterial hypertension is present in 74.46% of KTR patients with hypomagnesemia, with no statistically significant difference compared to those with normal magnesium levels. Additionally, 51.35% of hypertensive KTR patients in the 5–15 years post-Tx group and 62.07% of hypertensive KTR patients in the more than 15 years post-transplant group had hypomagnesemia. Dyslipidemia does not correlate with magnesium deficiency (*p* = 0.052). In the more than 15 years post-Tx KTR group, dyslipidemia was present in 61.11%, with a 54.54% incidence of magnesium deficiency.

While no significant associations were found with individual infections, magnesium deficiency was significantly associated with the number of various severe infections (*p* = 0.015). Specifically, KTR patients with hypomagnesemia exhibited a significantly higher incidence of more than two severe infections requiring hospitalization.

**Figure 3:**
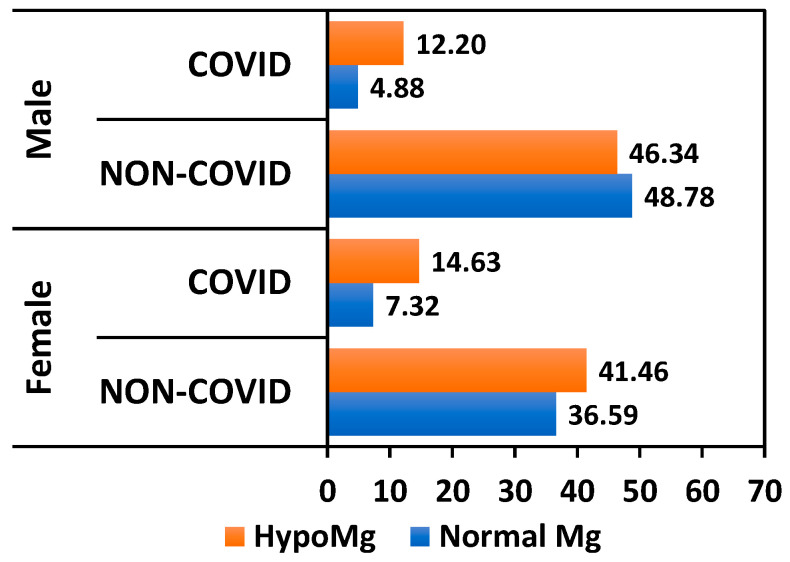
Relationship between the COVID-19 infection percentage and Mg levels.

COVID-19 infection was recorded in a higher percentage of KTRs with magnesium deficiency, in both men and women, though without statistical significance across the entire study cohort (*p* = 0.268). Conversely, among KTRs with normal serum magnesium levels, 7.32% of women and 4.88% of men contracted SARS-CoV-2 (Figure 3).

**Figure 4:**
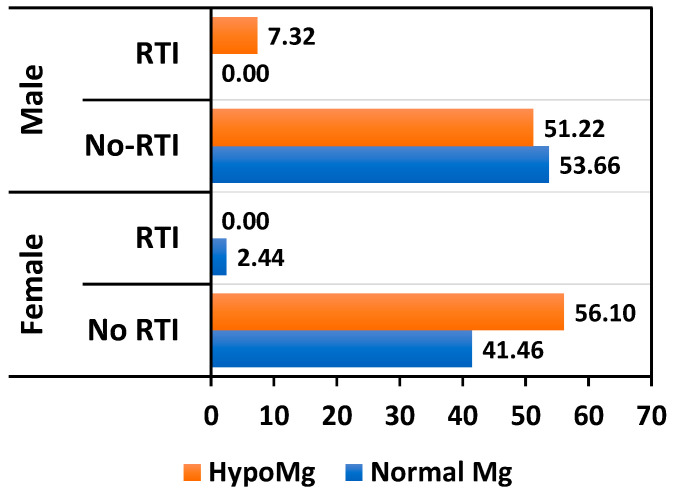
Relationship between non-COVID-19 respiratory tract infection percentages and Mg levels.

The incidence of non-COVID-19 respiratory infections requiring hospitalization was lower compared to SARS-CoV-2 infections. In female patients, the incidence was 2.44%, occurring exclusively in normomagnesemic KTRs due to magnesium supplementation. In contrast, the incidence in male patients was 7.32%, affecting only those with hypomagnesemia (Figure 4).

**Figure 5:**
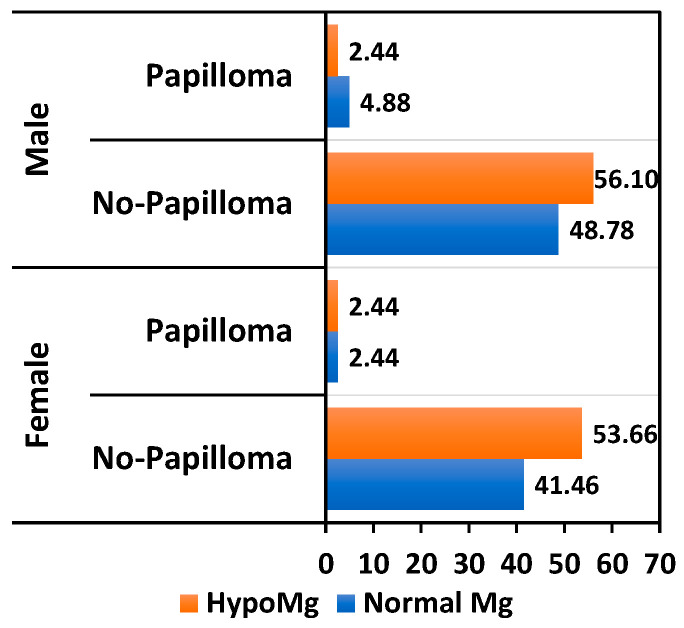
Relationship between HPV infection percentage and Mg level.

**Figure 6:**
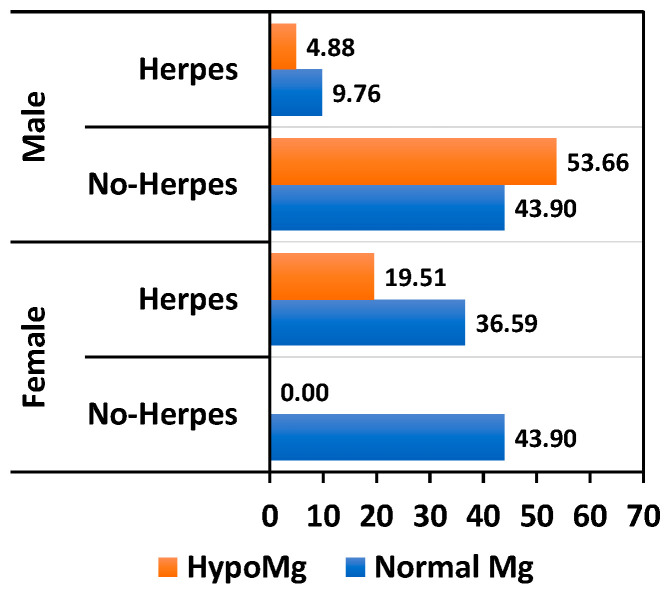
Relationship between Herpes virus infection percentage and Mg level.

**Figure 7:**
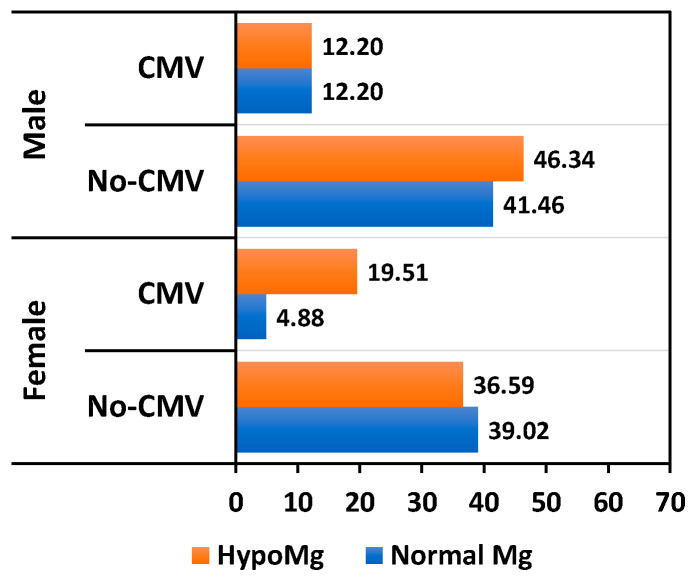
Relationship between CMV infection percentage and Mg level.

Although the presence of hepatitis B virus (HBV) infection does not directly correlate with hypomagnesemia, we showed that 50% of KTRs with HBV and 25% of KTRs with a papilloma virus infection exhibit magnesium deficiency. Warts had a low incidence in our cohort, but we noted double the prevalence in KTRs with normal Mg levels compared to hypomagnesemic patients (Figure 5). Herpes virus infections were common among KTRs, with a higher prevalence among female patients. Serum magnesium levels were normal in 36% of women and 9.7% of men (Figure 6). Although relatively common, the association between hypomagnesemia and CMV reactivation was not statistically significant. However, in our study, CMV reactivation had a higher incidence in KTRs with magnesium deficiency (27.65% vs. 17.5%, *p* = 0.31) (Figure 7).

### 3.3. The Effect of Magnesium Supplementation

**Figure 8:**
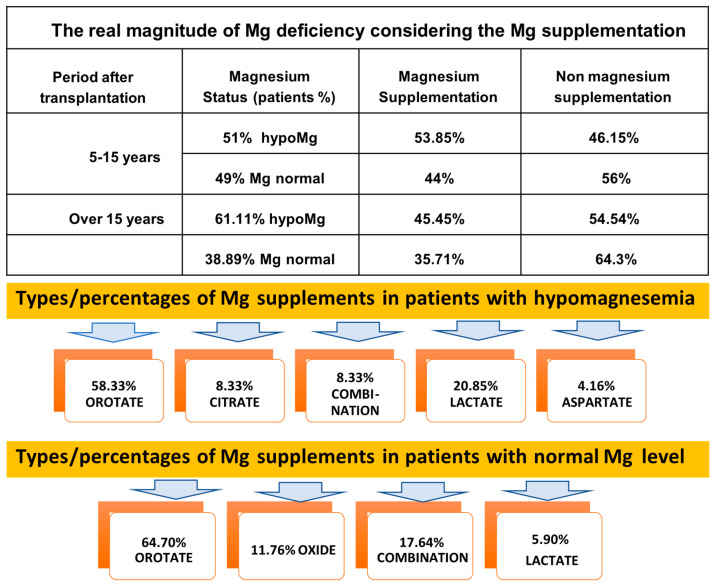
Magnesium supplementation products.

The correlation between serum magnesium levels and the use of exogenous magnesium supplementation is shown in Figure 8. Magnesium supplements are used by 47.12% of the KTRs in our cohort, either on their own or combined with other supplements. Despite magnesium being included in their therapeutic regimen, only 41.46% of these patients had serum magnesium levels within the normal range, while 58.54% showed magnesium deficiencies. Most KTRs use magnesium orotate, with an average dose of 32.8 mg/day. Over 90% of patients take a single daily dose of a magnesium supplement, regardless of type. When evaluating the two post-Tx periods separately, we noticed that over 50% of patients in the 5–15 years post-Tx group and over 45% in the over 15 years post-Tx group showed hypomagnesemia despite supplementation. Additionally, 44% of patients under 15 years post-Tx and 35% of those over 15 years post-Tx maintain normal magnesium levels due to daily oral magnesium use. Therefore, the true magnesium deficiency rate far exceeds the percentage of KTRs with clinically evident hypomagnesemia.

Despite supplementation, hypomagnesemia still occurs in 58.33% of patients using Mg orotate, 20.85% using magnesium lactate, 8.33% using magnesium citrate, 4.16% using magnesium aspartate, and 8.33% using various other available supplement combinations. In patients with normal serum magnesium levels, 64.7% use magnesium orotate, 11.7% use magnesium oxide, 5.9% use magnesium lactate, and 17.64% use various other magnesium combinations.

### 3.4. Risk Factors for Infections

We investigated potential risk factors in our patients for developing at least one viral infection or UTI using logistic regression. For this purpose, we used the binomial form of the glm function in R, with the results available in Table 3.

Both models failed to show a significant association between magnesium deficiency and the incidence of infections at a 0.05 threshold.

The odds of being diagnosed with a viral infection were higher in recipients of living donors (OR 4.23, 95%CI 1.04–21.05, *p* = 0.05), those with a lower level of proteins (OR 0.25, 95%CI 0.06–0.82, *p* = 0.029), and a lower GFR (OR 0.95, 95%CI 0.9–0.99, *p* = 0.05).

The odds of being diagnosed with an UTI were higher in the case of female patients (OR 6.25, 95%CI 1.56–25, *p* = 0.013) and those with a lower GFR (OR 0.94 95%CI 0.88–0.99, *p* = 0.046).

### 3.5. The Gender Disparity

Hypomagnesemia is significantly related to the number of different infections only in women (*p* = 0.006), who have a higher chance of developing at least two infections and a lower chance of not developing any (Table 4).

Similarly, hypomagnesemia is significantly associated with an increased chance of UTIs only in women (*p* = 0.03, Table 5).

## 4. Discussion

Kidney transplantation remains the best method for restoring renal function in patients with advanced chronic kidney disease. Three immunosuppressive lines are generally used in kidney transplant patients: CNIs (tacrolimus and cyclosporine), purine synthesis inhibitors (mycophenolate mofetil or azathioprine), and corticosteroids. CNIs are potentially nephrotoxic through multiple mechanisms, contributing, along with other factors, to the pathogenesis of chronic allograft nephropathy.

Immunosuppressive therapy is often associated with reduced serum magnesium levels, especially in KTRs receiving tacrolimus t and during the initial months post-Tx. However, the low post-Tx serum magnesium levels are due to multiple causes, specific to both kidney transplantation and more general factors: insufficient intake, metabolic acidosis, and treatment with diuretics or proton pump inhibitors [26]. Most studies note the persistence of hypomagnesemia for approximately two years post-Tx, after which serum magnesium levels may normalize or remain low in the long term [14,27].

### 4.1. Results of Demographic Evaluations Based on Post-Tx Interval

Our study included 87 KTRs within a period of immunological stability, at over 5 years post-Tx. Taking into account the unpredictable and relatively understudied dynamics of serum magnesium levels post-Tx, we performed the analysis separately in the 5–15 years post-transplant interval and also for over 15 years post-Tx, yielding two study groups. Demographic characteristics, laboratory analyses, and the presence of comorbidities were examined separately in these two groups.

As the results of comparative data for the two post-Tx intervals did not differ significantly regarding demographic data and laboratory tests, including serum magnesium levels (*p* = 0.65), the subsequent analyses focused predominantly on the total KTR cohort. Magnesium supplementation was provided to a similar percentage of patients (*p* = 0.52), supporting the decision to consider the patient cohort as a homogeneous group. Additionally, the incidence of infections, both viral and bacterial, more than 5 years post-Tx was consistent over time, reinforcing the notion of homogeneity in terms of immunosuppression stability beyond 5 years post-Tx. However, we noticed a higher percentage of KTRs who had discontinued corticosteroid therapy among those who were more than 15 years post-Tx.

### 4.2. Patient Characterization Based on the Presence or Absence of Hypomagnesemia

In our patients, hypomagnesemia was not correlated with age, sex, post-Tx duration, donor type, or underlying kidney disease. Some studies report a double incidence of magnesium deficiency in women compared to men, a finding not confirmed in our cohort [28,29]. Most of our KTRs received grafts from cadaveric donors (CD). It is expected that grafts from brain-dead donors, due to tubular changes induced by prolonged cold ischemia, would lead to a higher rate of urinary magnesium loss, which would be more evident in the early post-Tx period. Additionally, UKD did not significantly influence the incidence of post-Tx hypomagnesemia. This is explained by the fact that native kidneys are nonfunctional post-Tx, and their adjunctive role in additional magnesium loss is virtually inexistent.

KTRs with hypomagnesemia presented higher levels of inflammatory markers, such as CRP values. Animal and clinical studies support the hypothesis of a direct link between hypomagnesemia and inflammatory processes [30]. Magnesium influences the function of NK and CD8+ T cells, playing a role in regulating the proliferation and development of lymphocytes in general [31].

Numerous studies indicate a direct relationship between low magnesium levels and an increased risk of developing type 2 diabetes mellitus (T2DM) [28,29]. Variations in fasting glucose levels in relation to magnesium levels have been observed in KTRs, with those experiencing hypomagnesemia displaying higher blood glucose values [32,33]. Genetic involvement in the onset of NODAT has been demonstrated, which is linked to genetic variations in magnesium-modulated genes: TRPM6, SLC41A2, CLDN19, CNNM2, and FXYD2 [34,35,36]. Mitochondrial dysfunction has a central role in hypomagnesemia and NODAT; thus, it has been proposed that tacrolimus, a drug that directly affects the mitochondrial respiratory chain, is involved in diabetes onset among individuals with magnesium deficiency [37]. Although the existing literature supports the notion that hypomagnesemia may often trigger the onset of T2DM, our study found no correlation between hypomagnesemia and glycemic imbalance.

The negative effects of post-Tx hypomagnesemia on cardiovascular disease and the risk of developing T2DM are well-studied. Multiple in vivo and in vitro clinical studies highlight the association between low serum magnesium levels and post-Tx cardiovascular risk due to vascular calcifications, endothelial dysfunction, dyslipidemia, and inflammatory processes [38]. Furthermore, the mechanism of action of SGLT2 inhibitors may provide an integrative perspective on the relationship between serum glucose levels, serum magnesium levels, and endothelial dysfunction. The use of SGLT2 inhibitors leads to an increase in serum magnesium levels, an improvement in endothelial dysfunction, a reduction in arterial stiffness, and decreased resistance in the arterial circulation of the renal graft [39]. As most clinical trials involving SGLT2 inhibitors have excluded transplant populations, further data are needed to clarify their effects in post-Tx organ recipients.

The decline in glomerular filtration rate was observed more frequently, with statistical significance in KTRs with hypomagnesemia, representing both a cause and an effect of this condition. The relationship between hypomagnesemia and renal graft function has been studied, with a focus on the risk of graft loss in patients with cyclosporine nephropathy. Experimentally, hypomagnesemia induces chronic fibrotic lesions and glomerular dysfunction, while magnesium supplementation improves tubular atrophy and interstitial fibrosis. Magnesium supplementation also acts at the level of innate immunity, inhibiting monocyte and macrophage recruitment, partly through the reduction of chemotactic protein expression (osteopontin, monocyte chemotactic protein-1), fibrogenic molecules, and extracellular matrix proteins. Additionally, magnesium reduces the expression of endothelin-1 and decreases NFkB activation. These studies demonstrate a bidirectional relationship between serum magnesium levels and renal graft function [18,40].

### 4.3. Effects of Magnesium Supplementation Post-Transplant

Regarding our patient cohort, we would like to highlight several points. First, since there are patients with normal serum magnesium levels both without supplementation and following exogenous magnesium administration, the term ‘magnesium deficiency’ includes both those with hypomagnesemia and those with seemingly normal magnesium levels due to supplementation. Under these conditions, the true magnesium deficiency rate among our KTR cohort is, in fact, significantly higher.

Second, given the essential role of magnesium in the optimal functioning of the entire body, monitoring and maintaining serum magnesium levels within recommended limits is highly important. Due to the challenges in quantifying intracellular magnesium, reference values for total body magnesium are not yet established. Serum laboratory tests reflect only 1% of the body’s magnesium content, which is influenced by dietary intake, intestinal absorption, glomerular filtration, tubular reabsorption, and renal excretion [41,42]. The choice of magnesium supplements used to correct this deficiency is particularly important, given the diversity available on the market. Differences among these supplements are not solely related to magnesium dosage but also to bioavailability, which varies due to the use of different excipients and preparation technologies by different manufacturers. Magnesium bioavailability is higher in organic compounds compared to inorganic salts, which may also have gastrointestinal side effects. A daily supplementation of 200 mg of magnesium is generally sufficient to increase magnesium levels in the body, with treatment duration depending on the degree of hypomagnesemia. Studies in animal models indicate good absorption of magnesium from magnesium orotate, without gastrointestinal side effects [43,44,45]. Among the patients included in our study, 65.85% of the supplements used were magnesium orotate, with a much smaller proportion using other salts such as oxide, aspartate, lactate, or citrate. The high percentage of patients with serum magnesium deficiency who are using supplements raises questions regarding the bioavailability of magnesium in the administered supplements and the potential need for dose adjustment. The explanation for hypomagnesemia in patients using magnesium orotate, a compound with superior cellular bioavailability, may lie in the insufficient dose of only 32.8 mg/day. However, clarification of these aspects requires further studies.

### 4.4. Assessment of Risk Factors for Infections

Unlike infectious occurring in KTRs in the first months post-Tx, which are often pre-existing or nosocomially acquired, infections arising several years after transplantation are a consequence of immunosuppressive therapy. Infectious episodes represent a major cause of mortality in these patients, with risks linked to the degree of immunosuppression and the nature and clinical severity of the disease. In critical situations, with the acknowledged risk of graft rejection, reducing immunosuppressive therapy is a necessary approach.

The timing of infection diagnosis, often in the absence of specific symptoms, along with the implementation of an appropriate, personalized therapy for each patient, are the most important factors in infection management [46].

The statistical analysis of infection risk factors in our study reveals, alongside hypoproteinemia and reduced eGFR, an association between viral infections and kidney grafts from living donors. This finding contrasts with the existing literature and warrants further investigation using larger patient cohorts. As expected, for urinary tract infections, female sex is the primary risk factor, along with reduced eGFR. These associations are explained by the anatomy of the female urinary tract and the reduction in immunity concurrent with declining renal function. Neither magnesium deficiency nor magnesium supplementation had a statistically significant impact on the risk of infections. Surprisingly, NODAT was not identified as a risk factor for bacterial or viral infections in patients more than 5 years post-Tx.

We focused on CMV reactivation and the presence of HBV, HCV, papilloma, herpes virus, and COVID-19 infection within the spectrum of post-Tx viral infections. Our study revealed no significant increase in the incidence of CMV reactivation among KTRs experiencing hypomagnesemia. The reverse correlation—magnesium reduction secondary to CMV reactivation—may be attributed to tubulointerstitial injury to the renal graft, resulting in subsequent magnesium depletion, along with varying immunosuppressant concentrations due to the administration of antiviral therapy.

In our patient cohort, the incidence of SARS-CoV-2 infection is twice as high in KTRs with hypomagnesemia when compared to those with normal magnesium levels. COVID-19 infection induces cytotoxicity in renal epithelial cells and thrombotic microangiopathy [47,48]. Like CMV, this virus also acts by reducing the number of T lymphocytes and NK cells, thereby increasing the risk of secondary infections. This process results in immunosuppression and consequently the potential for associated infections [49]. Studies including the COVID-19-infected general population have demonstrated the beneficial effects of magnesium supplementation as a supportive therapy for these patients [50,51].

### 4.5. Advantages and Limitations of This Study

This study provides the advantage of presenting relevant data on the progression of pathology in kidney transplant recipients with hypomagnesemia 5 years after kidney transplantation. Most studies in this field focus on the immediate post-transplant period, characterized by significant fluctuations in laboratory results due to immunologic instability and frequent adjustments in therapeutic regimens. Generally, at 2 years post-transplant, serum magnesium levels tend to normalize; however, in our patients, hypomagnesemia persisted at a significant rate even 5 years post-transplant, a rate that is likely underestimated due to the frequent administration of magnesium supplements for correction.

In the setting of immunologic stability and a reduced need for immunosuppression, this study provides valuable insights into the role of magnesium on long-term outcomes of renal graft and kidney transplant recipient health. In this way, we attempt to fill an informational gap in the literature, and the data may support meta-analyses with significant statistical power.

The complexity of this study lies in the inclusion of magnesium supplements used by patients, whether medically recommended or not. These supplements have already been evaluated for bioavailability in in vitro and in vivo studies published in previous papers. The present study continues our long-standing interest in magnesium metabolism and the pharmacodynamics of magnesium supplements. Results obtained by inductively coupled plasma atomic emission spectrometry (ICP-AES) allowed us to evaluate the differences in bioavailability between different orally administered magnesium compounds [52].

There are several limitations to our study. First, there is the limitation inherent in observational trials that uncover associations but preclude the determination of cause–effect relationships. Second, this study was limited by being based on a single-center experience. Third, measuring serum magnesium levels and not intracellular magnesium levels may influence the assessment of the patients’ magnesium status. Thus, any conclusions drawn from the data must be replicated with a larger sample size and a prospective study design. Despite its limitations, this study provides significant data on a post-transplant population that has been minimally evaluated, offering information that may serve as a basis for future meta-analyses.

## 5. Conclusions

Our study results, within the context of its limitations, indicate an important relationship between low serum magnesium levels and infectious diseases in kidney transplant recipients who are over 5 years post-transplant. Immunosuppressive therapy promotes hypomagnesemia throughout the post-Tx period, necessitating ongoing serum magnesium level monitoring for these patients for the entire duration of graft survival. Oral magnesium supplementation may mitigate the onset or worsening of infectious, cardiovascular, or metabolic conditions. The administration of magnesium supplements is crucial for these patients, both during the immediate post-transplant phase and in the long term, with the dose and type of supplement customized for each individual.

## Figures and Tables

**Table 1:** Association between patient characteristics, laboratory results, infection diagnostics, and time elapsed since transplant.

		All Participants N = 87	5–15 Years After Transplant N = 51	More than 15 Years After Transplant N = 36	Statistical Test	*p*-Value
Demographics	Age	50.05	48.96	51.61	Wilcoxon	0.306
	Gender (female)	47.13%	41.18%	55.56%	Fisher	0.199
Donor type	Living	18	7	11	Fisher	0.066
Underlaying kidney disease	PKD	11 (12.64%)	9 (17.64%)	2 (5.55%)	Fisher	0.236
	TIN	9	4	3		
	Others	69	38	31		
Treatment	CNI	85	49	36	Fisher	0.509
	Corticosteroids	83 (95.4%)	51 (100%)	32 (88.88%)	Fisher	0.026
Laboratory results	PCR (0–5 mg/L)	6.23	6.8	5.42	Wilcoxon	0.756
	Glycemia (74–100 mg/dL)	97.95	100.29	94.64	Wilcoxon	0.2
	Creatinine (0.72–1.25 mg/dL)	1.43	1.41	1.47	Wilcoxon	0.646
	Total proteins (6.4–8.3 g/dL)	6.94	6.89	7.01	Wilcoxon	0.109
	eGFR	62.1	64.92	58.11	Wilcoxon	0.347
	Magnesium	1.62	1.63	1.6	Wilcoxon	0.652
Hypomagnesemia	YES	47 (54.02%)	25 (49.01%)	22 (61.11%)	Fisher	0.284
Magnesium supplements	YES	40 (45.97%)	25 (49.01%)	15 (41.66%)	Fisher	0.52
Comorbidity	Arterial hypertension	66 (75.86%)	37 (72.54%)	29 (80.55%)	Fisher	0.453
	Diabetes mellitus	11 (12.64%)	6 (11.76%)	5 (13.88%)	Fisher	0.756
	Dyslipidemia	42 (48.27%)	20 (39.21%)	22 (61.11%)	Fisher	0.052
Infections	HBV	7 (8.04%)	3 (5.88%)	4 (11.11%)	Fisher	0.441
	HCV	7 (8.04%)	1 (1.96%)	6 (16.6%)	Fisher	0.018
	UTI	32 (36.78%)	21 (41.17%)	11 (30.5%)	Fisher	0.37
	CMV	20 (22.98%)	13 (25.49%)	7 (19.4%)	Fisher	0.609
	Herpes	12 (13.79%)	5 (9.8%)	7 (19.4%)	Fisher	0.222
	Papilloma	5 (5.74%)	4 (7.84%)	1 (2.7%)	Fisher	0.398
	RTI	4 (4.59%)	3 (5.88%)	1 (2.7%)	Fisher	0.639
	Clostridium	1 (1.14)	1 (1.96%)	0	n/a	n/a
	COVID-19	16 (18.39%)	11 (21.56%)	5 (13.8%)	Fisher	0.412
Total different infections	0	26 (29.88%)	12 (23.52%)	14 (38.8%)	Fisher	0.103
	1	30 (34.48%)	22 (43.13%)	8 (22.2%)		
	2+	31 (35.63%)	17 (33.33%)	14 (38.8%)		

PKD = polycystic kidney disease, TIN = tubulointerstitial nephritis, CNI = calcineurin inhibitors, PCR = polymerase chain reaction, eGFR = estimated glomerular filtration rate, HBV = hepatitis B virus, HCV = C hepatitis C virus, UTI = Urinary tract infections, CMV = cytomegalovirus, RTI = respiratory tract infection.

**Table 2:** Association between patient characteristics, laboratory results, infection diagnostics, and magnesium deficiency.

Patient’s Characteristics		All Participants N = 87	Hypo- Magnesemia N = 47 (54.02%)	Normal Mg Level N = 40 (45.97%)	Statistical Test	*p*-Value
Demographics	Age	50.05	51.78	50.05	Wilcoxon	0.214
	Gender (male)	52.87	51.06%	55%	Fisher	0.829
Time since transplant	Years	13.93	14.78	12.92	Wilcoxon	0.252
Donor type	Living	18	10	8	Fisher	1
Underlying kidney disease	PKD	11	7	4	Fisher	0.733
	TIN	9	3	4		
	Others	69	37	32		
Treatment	CNI	85	47	38	Fisher	0.208
	Corticosteroids	83	44	39	Fisher	0.621
Laboratory results	PCR (0–5 mg/L)	6.23	7.4	4.86	Wilcoxon	0.012
	Glycemia (74–100 mg/dL)	97.95	93.51	103.17	Wilcoxon	0.112
	Creatinine (0.72–1.25 mg/dL)	1.43	1.54	1.31	Wilcoxon	0.028
	Total proteins (6.4–8.3 g/dL)	6.94	6.88	7.01	Wilcoxon	0.382
	eGFR	62.1	55.59	69.75	Wilcoxon	0.017
	Magnesium level (Mean)	1.62	1.48	1.78	n/a	n/a
Magnesium supplements	YES	40 (45.97%)	23 (48.9%)	17 (42.5%)	Fisher	0.666
Comorbidity	Arterial hypertension	66 (75.86%)	35 (74.47%)	31 (77.5%)	Fisher	0.805
	Diabetes mellitus	11 (12.64%)	6 (12.76%)	5 (12.5%)	Fisher	1
	Dyslipidemia	42 (48.27%)	20 (42.55%)	22 (55%)	Fisher	0.286
Infections	HBV	7 (8.04%)	2 (4.25%)	5 (12.5%)	Fisher	0.24
	HCV	7 (8.04%)	5 (10.64%)	2 (5%)	Fisher	0.444
	UTI	32 (36.78%)	21 (44.68%)	11 (27.5%)	Fisher	0.121
	CMV	20 (22.98%)	13 (27.65%)	7 (17.5%)	Fisher	0.313
	Herpes	12 (13.79%)	8 (17.02%)	4 (10%)	Fisher	0.534
	Papilloma	5 (5.74%)	2 (4.25%)	3 (7.5%)	Fisher	0.657
	RTI	4 (4.59%)	3 (6.38%)	1 (2.5%)	Fisher	0.621
	Clostridium	1 (1.15%)	0	1 (2.5%)	n/a	n/a
	COVID-19	16 (18.39%)	11 (23.4%)	5 (12.5%)	Fisher	0.268
Total different infections	0	26 (29.88%)	12 (25.53%)	14 (35%)	Fisher	0.015
	1	30 (34.48%)	12 (23.53%)	18 (45%)		
	2+	31 (35.63%)	23 (48.93%)	8 (20%)		

PKD = polycystic kidney disease, TIN = tubulointerstitial nephritis, CNI = calcineurin inhibitors, PCR = polymerase chain reaction, eGFR = estimated glomerular filtration rate, HBV = hepatitis B virus, HCV = hepatitis C virus, UTI = Urinary tract infections, CMV = cytomegalovirus, RTI = respiratory tract infection; n/a = not applicable.

**Table 3:** Multivariate logistic regression for the incidence of Viral infections and UTIs.

		All Participants N = 87	Viral Infections (Yes) N = 52				UTI (Yes) N = 32			
		Mean	OR	95% CI Interval		*p*-Value	OR	95% CI Interval		*p*-Value
Demographics	Age	50.05	0.99	0.93	1.04	0.658	0.98	0.92	1.04	0.538
	Gender (female)	41	0.75	2.63	0.22	0.64	6.25	1.56	25.00	0.013
Donor type	Living	18	4.23	1.04	21.05	0.05	0.43	0.07	2.28	0.34
Time since transplant	5–15 years	51	0.66	0.2	2.11	0.489	0.41	0.08	1.75	0.242
Underlaying kidney disease	PKD	11	1.71	0.49	6.61	0.411	0.5	0.1	2.34	0.381
	TIN	9								
	Others	69								
Laboratory results	CRP (0–5 mg/L)	6.23	0.94	0.87	1.01	0.109	1.02	0.95	1.11	0.631
	Glycemia (74.00–100.00 mg/dL)	97.95	1.01	0.97	1.04	0.698	1.02	0.98	1.05	0.385
	Creatinine (0.72–1.25 mg/dL)	1.43	0.17	0.02	1.17	0.084	0.13	0.01	1.28	0.1
	Total proteins (6.4–8.3 g/dL)	6.94	0.25	0.06	0.82	0.029	0.29	0.06	1.2	0.101
	eGFR	62.1	0.95	0.9	0.99	0.05	0.94	0.88	0.99	0.046
Hypomagnesemia	YES	47	0.62	0.2	1.84	0.394	0.43	0.11	1.53	0.203
Magnesium supplements	YES	40	1.46	0.51	4.32	0.482	0.36	0.1	1.19	0.101
Comorbidity	Arterial hypertension	66	0.82	0.23	2.93	0.756	0.97	0.2	4.57	0.974
	Diabetes mellitus	11	0.3	0.04	1.94	0.224	0.89	0.1	8.94	0.921
	Dyslipidemia	42	1.19	0.39	3.58	0.752	1.09	0.27	4.35	0.98

PKD = polycystic kidney disease, TIN = tubulointerstitial nephritis, CNI = calcineurin inhibitors, CRP = C-reactive protein, eGFR = estimated glomerular filtration rate.

**Table 4:** Association between magnesium deficiency and the number of infections for each gender.

Gender	Number of Infections	All Participants	Hypomagnesemia	Normal Magnesium Level	Statistical Test	*p*-Value
		N = 87	N = 47	N = 40		
Female	0	11	3	8	Fisher	0.006
	1	10	4	6		
	2+	20	16	4		
Male	0	15	9	6	Fisher	0.387
	1	20	8	12		
	2+	11	7	4		

**Table 5:** Association between magnesium deficiency and the presence of UTIs for each gender.

Gender	All Participants	Diagnostic	Hypomagnesemia	Normal Magnesium Level	Statistical Test	*p*-Value
	N = 87		N = 47	N = 40		
Female	41	UTI	17	7	Fisher	0.03
		no-UTI	6	11		
Male	46	UTI	4	4	Fisher	0.999
		no-UTI	20	18		

UTI = Urinary tract infections.

## Data Availability

The data presented in this study are available on request from the corresponding author. The data are not publicly available due to ethical reasons.
